# Microscopic and Color Changes in Direct Dental Restorative Composite Resins upon Immersion in Beverages: Characterization by Scanning Electron Microscopy (SEM) and Energy-Dispersive X-ray Spectroscopy (EDS)

**DOI:** 10.3390/biomedicines12081740

**Published:** 2024-08-02

**Authors:** Adrian Ioan Hajdu, Ramona Dumitrescu, Octavia Balean, Daniela Jumanca, Ruxandra Sava-Rosianu, Lucian Floare, Vanessa Bolchis, Titus Vlase, Atena Galuscan

**Affiliations:** 1 Translational and Experimental Clinical Research Centre in Oral Health, Department of Preventive, Community Dentistry and Oral Health, “Victor Babes” University of Medicine and Pharmacy, 300040 Timisoara, Romania; hajdu.adrian@umft.ro (A.I.H.); dumitrescu.ramona@umft.ro (R.D.); jumanca.daniela@umft.ro (D.J.); sava-rosianu.ruxandra@umft.ro (R.S.-R.); floare.lucian@umft.ro (L.F.); vanessa.bolchis@umft.ro (V.B.); galuscan.atena@umft.ro (A.G.); 2 Department I, Department of Preventive, Community Dentistry and Oral Health, “Victor Babes” University of Medicine and Pharmacy, Eftimie Murgu Sq. No 2, 300041 Timisoara, Romania; 3 Research Centre for Thermal Analysis in Environmental Problems-ICAM, West University of Timisoara, Pestalozzi Street 16, 300115 Timisoara, Romania; titus.vlase@e-uvt.ro

**Keywords:** dental resin composite, SEM analyses, EDS analyses, color stability, CIELAB

## Abstract

This study aimed to evaluate the staining sensitivity and surface changes in recent composite resins (Herculite Ultra XRV (Kerr, Bolzano, Italy), G-ænial A’CHORD (GC Corp, Tokyo, Japan), and Omnichroma (Yamaguchi, Japan)) when exposed to common beverages such as coffee, red wine, and Coca-Cola. A total of 60 disk-shaped specimens were prepared from three different resin composites (n = 20 each). The specimens were exposed to coffee, red wine, and Coca-Cola for 10 days. Color measurements were taken using a spectrophotometer, and surface morphology and elemental composition were analyzed using SEM and EDS. The SEM and EDS analyses revealed significant changes in the surface morphology and elemental composition of the composites after immersion. Coffee and wine caused significant surface degradation, whereas Coca-Cola resulted in the greatest degree of surface and elemental variations. Color changes (ΔE = 4 ± 0.52) were most notable in Coca-Cola for Herculite Ultra XRV (Kerr, Italy), in red wine for G-ænial A’CHORD (GC Corp, Japan) (ΔE = 12.51 ± 0.38), and in coffee for Omnichroma (Yamaguchi, Japan) (ΔE = 10.85 ± 1.03). The tested beverages significantly affected both the surface condition and the chemical composition of the resin at the surface level. These findings highlight the importance of understanding the effects of common dietary beverages on dental composites.

## 1. Introduction

Dental composite resins are commonly used in dental practices due to their excellent esthetic properties and ability to bond to dental surfaces. These resins differ in their matrix composition, particle size, filler type, and volume. With recent advancements in technology, dental restoration materials have significantly improved in terms of durability and esthetic results [1]. Among the array of restorative materials available, resin-based options have consistently demonstrated superior clinical outcomes. Resin composites are exemplary choices for various clinical applications, thanks to their strong adhesive bonding, pleasing esthetics, translucency, mechanical strength, and biocompatibility [2,3].

In recent years, dentists have increasingly preferred ceramic and resin-containing restorative materials for tooth restoration to meet patients’ esthetic expectations. Resin-based restorative materials are particularly popular because they easily adapt to the natural color of teeth, fulfilling esthetic requirements effectively [4,5].

With the use of nano technology in dentistry, manufacturers are introducing composite resins with single-shade systems instead of more complex color systems. It is stated that these composite resins consisting of nanofillers (nanomer) and nanomer groups (nanocluster) provide more effective color harmony with dental tissues because of their “chameleon effect” properties. Recently, single-shade composite resin, which can be used for all tooth shades, has become available for dentists [5,6]. The biggest advantage of the one-shade composite resin system is that it allows for restoration consistent with the tooth color in a short time without determining the tooth shade in esthetic restorations [5].

The degradation of composite materials remains a significant problem in dental practice, despite technological advances and improvements in the physical and mechanical properties of dental composites. Various beverages can cause the surface erosion of dental composites. This raises concerns about the erosive potential of these beverages on dental tissues and the long-term integrity of restorative materials. While modern society promotes a healthier lifestyle that improves patients’ quality of life, it also offers a wide range of beverages and foods that can have a negative impact on the body’s overall balance and oral health [7].

The ability of restorative dental materials to withstand occlusal forces and exposure to various substances in the mouth is crucial for their long-term clinical performance. Chemical factors that can adversely affect these materials include low pH from cariogenic biofilm, consumption of acidic drinks and foods, and the action of enzymes. These factors can soften and roughen the outermost layers of restorative materials, compromising their durability and effectiveness over time [8].

Composites are intricate heterogeneous materials consisting of a resin base embedded with filler particles that are coated with a bonding agent. These particles vary in origin, size, and shape. Consequently, the surface of these materials can exhibit a heterogeneous interface of particles distributed across different physical and chemical phases. As a result, commercially available restorative composite resins will display varying surface roughness and polishability depending on the type of filler used.

Most resin–matrix composites consist of methacrylate-based monomers (organic matrices), such as bisphenol A diglycidyl methacrylate (Bis-GMA), ethoxylated bisphenol A diglycidyl methacrylate (Bis-EMA), urethane dimethacrylate (UDMA), and triethylene glycol dimethacrylate (TEGDMA). These composites also include inorganic filler particles (dispersed phase), photoinitiator systems, and various minor additives such as stabilizers and pigments [9]. Resin–matrix composites are typically classified based on the size and type of their inorganic filler particles, ranging from macro- to nano-scale dimensions. The color stability of these materials is influenced by their chemical composition, including the organic matrix, the content of inorganic particles, and the type of photoinitiator system. A higher filler content results in a reduced proportion of the organic matrix [10].

Today, resin–matrix composites enable a more conservative approach to biomimetic rehabilitation and have consequently become the preferred choice for chairside restorative materials [11].

The color change in esthetic restorative materials is a primary reason for their replacement. The aging process in the oral environment is influenced by various extrinsic and intrinsic factors; extrinsic factors are related to the environment to which the materials are exposed, while intrinsic factors are linked to the chemical composition of the materials [9].

Teeth coloration results from both intrinsic and extrinsic factors. Intrinsic color pertains to the light absorption and scattering within enamel and dentin, while extrinsic color arises from external substances (e.g., tea, red wine, chlorhexidine, iron salts) absorbed onto the enamel surface and pellicle coating, leading to extrinsic staining [12].

The motivation for this study stemmed from a clear clinical reality: composite fillings are predisposed to deterioration, affecting the functionality of the dental system, with the degree of deterioration varying according to the location of the filling. An important consideration in our study was the negative impact that many commonly consumed beverages have on oral health.

This study aimed to evaluate the staining sensitivity and surface changes in recent composite resins (Herculite Ultra XRV, G-ænial A’CHORD, and Omnichroma) when exposed to common beverages such as coffee, red wine, and Coca-Cola. Using Scanning Electron Microscopy (SEM) and Energy-Dispersive X-ray Spectroscopy (EDS), this research study aimed to investigate how these beverages impact the color stability and surface integrity of these dental materials over specified immersion periods.

## 2. Materials and Methods

### 2.1. Sampling Preparations

In this study, a total of 60 disk-shaped specimens were prepared from three different resin composites. Each composite material had 20 specimens, with 5 kept as controls, 5 immersed in coffee, 5 in wine, and 5 in Coca-Cola. The specimens were created using metallic molds with dimensions of 8 mm × 2 mm to match the polymerization units’ diameter. The specimens were then exposed from the top to light using a dentist wireless LED curing light (1200 mW, USA, 3M Espe Elipar S10, Dental Products, St. Paul, MN, USA) at a distance of approximately 1 mm from the surface. The irradiance of the curing light was evaluated using the Bluephase Meter II (Ivoclar) following the manufacturer’s instructions, ensuring the irradiance was consistently 1200 mW/cm^2^. The curing time was set to 40 s based on findings in the literature [1,13], which suggest this duration ensures the complete photopolymerization of the composites. This extended curing time was chosen to ensure that all layers of the material were fully cured, considering potential variations in light transmission through the composite material.

After the polymerization was completed, both surfaces of all samples were polished with polishing disks (Optidisc Polishing Discs, Sds Kerr Danbury, CT, USA) for a period of 60 s at a low speed, with mild pressure, clinical contra-angle, and micromotor. To ensure standardization, the polishing process was carried out by a single operator using a low-speed hand device, applying uniform, dry, and intermittent pressure [14]. The Kappa value for the intra-examiner calibration was 0.8, confirming that the operator’s performance was reliable and thereby validating the sampling preparations. Subsequently, all specimens were subjected to ultrasonic cleaning in distilled water for 5 min after rinsing. To ensure consistency, the same operator carried out all procedures. The specimens were grouped based on the type of dental resin used.

Group 1—Herculite Ultra XRV (Kerr, Italy), which is a universal nanohybrid composite featuring filler technology with barium glass fillers averaging 0.4 µm in particle size, along with silica nanoparticles ranging from 20 to 50 nm [15] (Table 1).

Group 2—G-ænial A’CHORD (GC), a hybrid composite, combines two types of pre-polymerized resin fillers. These fillers, incorporating microfillers into a resin matrix, are then milled into particles averaging 16 to 17 μm in size. This composite offers both clinical radiopacity and esthetic appeal [16] (Table 1).

Group 3—Omnichroma (Tokuyama), which is designed for use in most direct restorative clinical cases. Omnichroma is a universal single-color structural composite and its components include UDMA and TEGDMA, along with supra-nano spherical fillers. These fillers comprise silica–zirconia particles averaging 0.3 μm in size, ranging from 0.2 to 0.6 μm [17] (Table 1).

### 2.2. Adherence of Red Wine, Black Coffee and Coca-Cola

To investigate color variations caused by various beverages, samples of each composite resin (n = 15 each) were immersed in coffee, wine, and cola. A control group (n = 5) was included. All specimens were kept in an incubator at 37 °C for 10 days, with the staining agents replaced daily to prevent bacterial or yeast contamination [18]. The plates were submerged in black coffee (prepared with 5 g of coffee to 150 mL boiled water, Nespresso), red wine (Budureasca Clasic, Feteasca Neagra, Dealu Mare, Romania), and Coca-Cola for 20 min each day over 10 consecutive days. After the daily immersion period, the specimens were stored in a humid environment within an incubator maintained at 37 °C for the remainder of the 24 h cycle. This setup aimed to mimic the oral environment, which is generally moist. The humid environment was maintained by placing the specimens in a sealed container with a small amount of distilled water to ensure constant humidity. The control group specimens were maintained in a moist condition similar to the experimental groups, except they were not immersed in any beverage.

### 2.3. Color Assessment

The spectrophotometer (Vita Easyshade^®^ V Compact Vita, Zahnfabrik, Bad Sackingen, Germany) was used for color measurements [19]. The photometer was calibrated in accordance with the National Institute of Standards and Technology (NIST) tiles. The setting at which the tests were run included a 10 nm wavelength interval, a 360 to 750 nm spectral range, and a 45 reflectance angle. The background was black and an average of three scans were performed for each specimen. The CIELAB color system was employed, comprising L*, a*, and b* axis identification. Here, “L” ranged from 0 to 100 (brightness to darkness), the “a” axis represented red to green colors (90 to 70 range value), and the “b” axis represented yellow to blue colors (coordinate value range from 80 to 100) [2]. The color change ΔE was calculated using the following equation:

ΔE = (ΔL^2^ + Δa^2^ + Δb^2^)^1/2^

∆E values greater than 2.7 indicate “very distinct” changes, values between 1.2 and 2.7 indicate “distinct” changes, and values less than 1.2 indicate “non-distinct” changes [12]. The measurements were taken on days 4 and 10. Before and after each immersion session, the specimens were gently rinsed with distilled water to remove any residual beverage and air-dried. This ensured that the color measurements reflected changes in the composite material itself rather than surface residues from the beverages.

### 2.4. SEM and EDS Analyses

Scanning Electron Microscopy (SEM) analysis was performed with the Inspect scanning electron microscope in low vacuum, at a pressure of 60 Pa and 30 kV voltage, with a scale bar of 50 µm. Energy-Dispersive X-ray Spectroscopy (EDS) analysis was performed with the -JSM IT 200 (JEOL Ltd., Tokyo, Japan) electron microscope in low vacuum 80 Pa, landing voltage 15.0 kV, and magnification at ×500. EDS analysis was conducted to evaluate the elemental composition of the specimens. EDS was utilized to identify and quantify the elemental changes that occurred in the resin composites after immersion in the different solutions. This analysis offered insights into the surface and chemical alterations of the resins, supplementing the visual assessment of color changes.

### 2.5. Statistical Analysis

To compare the mean data across different levels of each variable, we used multivariate analysis of variance (MANOVA) with SPSS version 23. This statistical test was applied to each independent variable to determine which dependent variables varied with the levels of the independent variable. In other words, it helped us see how each manipulated sample, depending on the composite, differed at 4 and 10 days of experimental manipulation. Additionally, we used Student’s *t*-test to test whether the difference between the response of two groups was statistically significant or not.

## 3. Results

### 3.1. SEM and EDS Analyses

The materials evaluated in this study—Herculite Ultra XRV (Kerr, Italy), G-ænial A’CHORD (GC Corp, Japan), and Omnichroma (Yamaguchi, Japan)—were immersed in coffee, red wine, and Coca-Cola, chosen due to their common consumption and potential impact on dental restorations.

**Figure 1:**
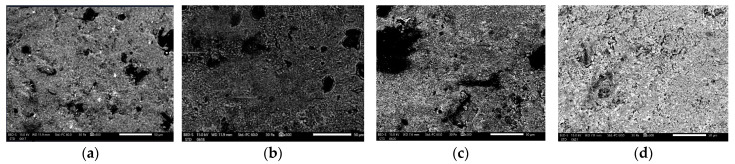
Scanning Electron Microscopy images of (**a**) control Herculite Ultra XRV (Kerr, Italy) (**b**) Herculite Ultra XRV (Kerr, Italy) immersed in coffee for 10 days, (**c**) Herculite Ultra XRV (Kerr, Italy) immersed in wine for 10 days, (**d**) Herculite Ultra XRV (Kerr, Italy) immersed in Coca-Cola for 10 days at a magnification of ×500.

The control sample of Herculite Ultra XRV (Kerr, Italy) exhibited a relatively smooth surface with minimal defects, indicating an intact and uniform composite structure under neutral conditions. In contrast, immersion in coffee resulted in increased surface roughness with small pits and cracks, suggesting that the coffee components interact with the composite material and initiate degradation. The sample immersed in wine showed significant surface degradation, with more pronounced cracks and noticeable material loss, indicating a stronger corrosive effect compared to coffee. The sample exposed to Coca-Cola demonstrated extensive surface roughness, large pits, and significant material degradation, reflecting the highly corrosive nature of Coca-Cola, likely due to its acidic content and other reactive components (Figure 1).

The EDS analysis of the Herculite Ultra XRV (Kerr, Italy) composite indicates that after 10 days of immersion, the elemental composition changed as follows: in coffee, carbon decreased from 33.69 ± 2.32% to 21.68 ± 0.36% and oxygen decreased from 40.16 ± 3.09% to 33.66 ± 0.50%; in red wine, carbon decreased to 8.77 ± 0.23% and oxygen decreased to 8.15 ± 0.51%; in Coca-Cola, carbon decreased to 10.68 ± 0.20% and oxygen decreased to 32.55 ± 0.37%. Additionally, silicon, initially at 15.43 ± 1.31%, decreased to 12.37 ± 0.19% in Coca-Cola, while sulfur increased from 1.28 ± 0.09% to 7.20 ± 0.14% in Coca-Cola immersion. Barium showed a significant increase from 9.72 ± 0.39% to 34.12 ± 0.58% after 10 days of Coca-Cola immersion (Table 2).

The SEM and EDS analyses reveal that immersion in coffee, wine, and Coca-Cola resulted in notable changes in the surface morphology and elemental composition of the composite material. Coffee and wine caused more pronounced surface degradation and increased the presence of elements not originally in the control sample, such as Ba and Yb. Coca-Cola, known for its acidic properties, led to the highest degree of surface roughness and elemental variation, indicating severe interaction with the composite material.

**Figure 2:**
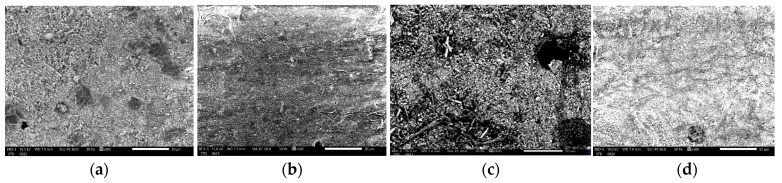
Scanning Electron Microscopy of (**a**) control G-ænial A’CHORD (GC Corp, Japan), (**b**) G-ænial A’CHORD (GC Corp, Japan), immersed in coffee for 10 days, (**c**) G-ænial A’CHORD (GC Corp, Japan), immersed in wine for 10 days, (**d**) G-ænial A’CHORD (GC Corp, Japan), immersed in Coca-Cola for 10 days at a magnification of ×500.

The SEM analysis of G-ænial A’CHORD (GC Corp, Japan), revealed distinct changes in the surface morphology among the samples. The control group exhibited a relatively uniform surface with minor irregularities. In contrast, the coffee-immersed sample showed noticeable surface roughness and deposits, likely from organic compounds in the coffee. The wine-immersed sample displayed significant surface irregularities and a higher degree of porosity. The Coca-Cola-immersed sample had a highly irregular surface with a substantial amount of surface deposits (Figure 2).

The sample immersed in Coca-Cola showed the highest carbon content, likely due to the beverage’s high sugar content, which decomposes to carbon during analysis. The wine-immersed sample had the highest oxygen content, suggesting the presence of oxidizing components from the wine, and contained unique elements such as calcium and strontium, indicating significant compositional alterations. The coffee-immersed sample displayed a similar aluminum content to the control but had the highest potassium concentration among the experimental groups (Table 2). Overall, immersion in coffee, wine, and Coca-Cola resulted in distinct changes in elemental compositions, with increased carbon content in coffee and Coca-Cola samples and unique elemental additions in the wine sample. These findings highlight how different solutions can significantly alter the surface chemistry of materials, which is crucial for understanding their interaction mechanisms in various environments.

**Figure 3:**
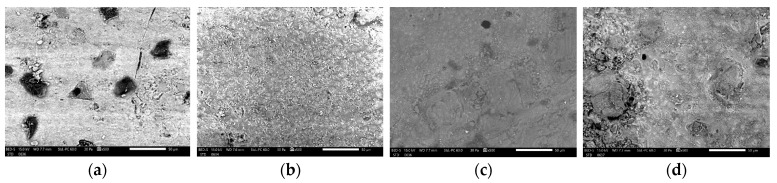
Scanning Electron Microscopy of (**a**) control Omnichroma (Yamaguchi, Japan), (**b**) Omnichroma (Yamaguchi, Japan) immersed in coffee for 10 days, (**c**) Omnichroma (v) immersed in wine for 10 days, (**d**) Omnichroma (Yamaguchi, Japan) immersed in Coca-Cola for 10 days at a magnification of ×500.

The SEM and chemical analysis of the Omnichroma (Yamaguchi, Japan) composite under different conditions revealed significant variations in both surface morphology and elemental composition. The control sample exhibited a relatively smooth surface with minimal defects, indicating an intact and uniform composite structure under neutral conditions. In contrast, the sample immersed in coffee showed increased surface degradations with small pits and cracks, suggesting degradation initiated by coffee components. The sample immersed in wine displayed moderate surface irregularities and minor erosions, indicating mild interaction between the wine and the composite’s surface (Figure 3). The sample immersed in Coca-Cola demonstrated significant surface roughness and noticeable erosion, indicating a strong degradative effect of the acidic beverage. Chemically, the carbon content increased notably in the sample immersed in Coca-Cola, indicating substantial organic residue deposition or surface changes. Oxygen and silicon content also varied across the samples, reflecting the impact of different immersion solutions on the composite material’s chemical stability. Specifically, the control sample contained 6.90 ± 0.22% carbon and 43.16 ± 0.47% oxygen, while the sample immersed in Coca-Cola had 65.44 ± 0.37% carbon and 24.70 ± 0.48% oxygen. Other elemental contents such as sodium, zirconium, aluminum, sulfur, and calcium also exhibited changes, further illustrating the different levels of interaction between the composite material and the various immersion solutions (Table 2).

The EDS analysis of Herculite Ultra XRV (Kerr, Italy), Gaenial A’CHORD (GC Corp, Japan), and Omnichroma (Yamaguchi, Japan) composites after 10 days of coffee immersion revealed differing degrees of elemental composition changes. Herculite Ultra XRV (Kerr, Italy) showed a significant reduction in carbon from 33.69 ± 2.32% to 21.68 ± 0.36% and in oxygen from 40.16 ± 3.09% to 33.66 ± 0.50%. Gaenial A’CHORD (GC Corp, Japan) experienced a notable decrease in carbon from 39.49 ± 2.20% to 34.82 ± 0.85% and a dramatic drop in oxygen from 31.89 ± 2.45% to 11.37 ± 0.70%, as well as a decrease in aluminum from 6.08 ± 0.65% to 3.20 ± 0.26%. Omnichroma (Yamaguchi, Japan), on the other hand, saw an increase in carbon from 6.90 ± 0.22% to 27.14 ± 0.49% and a decrease in oxygen from 43.16 ± 0.47% to 33.06 ± 0.58%, alongside a reduction in aluminum from 0.52 ± 0.04% to 0.36 ± 0.05% (Table 2).

Comparatively, Gaenial A’CHORD (GC Corp, Japan) appears to be the most affected by coffee immersion, with the most significant reductions in both oxygen and aluminum content. The SEM images further corroborated these findings, showing more pronounced surface morphology changes in Gaenial A’CHORD (GC Corp, Japan) compared to Herculite Ultra XRV (Kerr, Italy) and Omnichroma (Yamaguchi, Japan), indicating a higher susceptibility to coffee-induced degradation (Table 2).

The EDS analysis of Herculite Ultra XRV (Kerr, Italy), Gaenial A’CHORD (GC Corp, Japan), and Omnichroma (Yamaguchi, Japan) composites after 10 days of red wine immersion showed varied impacts on their elemental compositions. Herculite Ultra XRV (Kerr, Italy) exhibited a significant decrease in carbon from 33.69 ± 2.32% to 8.77 ± 0.23% and in oxygen from 40.16 ± 3.09% to 8.15 ± 0.51%. Gaenial A’CHORD (GC Corp, Japan) also showed notable reductions, with carbon dropping from 39.49 ± 2.20% to 28.52 ± 0.54% and oxygen from 31.89 ± 2.45% to 44.98 ± 1.08%, alongside a reduction in silicon from 22.53 ± 1.28% to 6.34 ± 0.26%. Omnichroma (Yamaguchi, Japan), however, saw an increase in carbon from 6.90 ± 0.22% to 25.13 ± 0.55% and a decrease in oxygen from 43.16 ± 0.47% to 25.55 ± 0.55%, along with an increase in silicon from 36.36 ± 0.39% to 37.55 ± 0.39% (Table 2).

Comparatively, Herculite Ultra XRV (Kerr, Italy) was the most affected by red wine immersion, with the highest reduction percentages in carbon and oxygen, indicating significant degradation. SEM images further illustrated these findings for Herculite Ultra XRV (Kerr, Italy), Gaenial, and Omnichroma (Yamaguchi, Japan). The surface morphology of Herculite Ultra XRV (Kerr, Italy) showed the most pronounced changes, corroborating its higher susceptibility to red wine-induced degradation.

Among the three dental composites—Herculite Ultra XRV (Kerr, Italy), Gaenial A’CHORD (GC Corp, Japan), and Omnichroma (Yamaguchi, Japan)—subjected to immersion in Coca-Cola, Omnichroma (Yamaguchi, Japan) exhibited the most significant compositional modifications. The EDS analysis of Herculite Ultra XRV (Kerr, Italy), Gaenial A’CHORD (GC Corp, Japan), and Omnichroma (Yamaguchi, Japan) composites after 10 days of Coca-Cola immersion revealed significant changes in their elemental compositions. Herculite Ultra XRV (Kerr, Italy) showed a decrease in carbon from 33.69 ± 2.32% to 10.68 ± 0.20% and in silicon from 15.43 ± 1.31% to 12.37 ± 0.19%, with an increase in sulfur from 1.28 ± 0.09% to 7.20 ± 0.14% and in barium from 9.72 ± 0.39% to 34.12 ± 0.58%. Gaenial A’CHORD (GC Corp, Japan) experienced a substantial rise in carbon from 39.49 ± 2.20% to 53.09 ± 1.84% and a decrease in silicon from 22.53 ± 1.28% to 16.06 ± 0.89%. Omnichroma (Yamaguchi, Japan) saw an increase in carbon from 6.90 ± 0.22% to 65.44 ± 0.37% and a reduction in silicon from 36.36 ± 0.39% to 6.33 ± 0.13% (Table 2).

Comparatively, Omnichroma (Yamaguchi, Japan) was the most affected by Coca-Cola immersion, with the highest increase in carbon and the most significant decrease in silicon, indicating substantial compositional alterations. The SEM images provided visual confirmation of the fact that the surface morphology changes were the most pronounced in Omnichroma (Yamaguchi, Japan), corroborating its higher susceptibility to degradation from Coca-Cola immersion. These results highlight the importance of considering the impact of acidic beverages on the durability and integrity of dental composites.

### 3.2. Color Metrics and the Impact of Various Substances (Red Wine, Black Coffee, and Coca-Cola)

**Figure 4:**
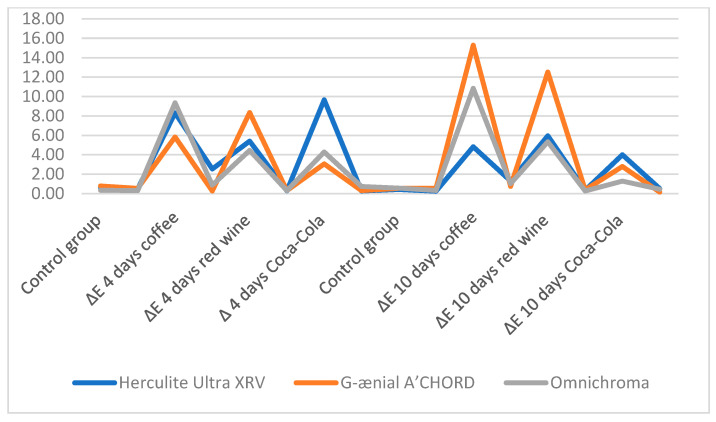
Comparison of the ΔE among the beverages and dental resins.

The color change (ΔE) results for Herculite Ultra XRV (Kerr, Italy), Gaenial A’CHORD (GC Corp, Japan), and Omnichroma (Yamaguchi, Japan) composites after immersion in coffee, red wine, and Coca-Cola over 4 and 10 days showed varying degrees of discoloration. After 4 days of immersion, Herculite Ultra XRV (Kerr, Italy) exhibited the highest ΔE in Coca-Cola (9.66 ± 0.28) and the lowest in red wine (5.40 ± 0.29). Gaenial A’CHORD (GC Corp, Japan) showed the highest discoloration in red wine (8.35 ± 0.30) and the lowest in Coca-Cola (3.06 ± 0.31). Omnichroma (Yamaguchi, Japan) presented the highest ΔE in coffee (9.36 ± 0.30) and the lowest in red wine (4.46 ± 0.30). After 10 days, Herculite Ultra XRV (Kerr, Italy) had a significant discoloration in red wine (5.96 ± 0.35) and the lowest in Coca-Cola (4.00 ± 0.52). Gaenial A’CHORD (GC Corp, Japan) experienced the highest ΔE in coffee (15.28 ± 0.74) and the lowest in Coca-Cola (2.80 ± 0.16). Omnichroma (Yamaguchi, Japan) showed the most considerable color change in coffee (10.85 ± 1.03) and the least in Coca-Cola (1.28 ± 0.45). These results indicate that Gaenial A’CHORD (GC Corp, Japan) was the most affected by coffee immersion, while Herculite Ultra XRV (Kerr, Italy) and Omnichroma (Yamaguchi, Japan) showed significant changes across all solutions, with varying degrees of sensitivity (Table 3, Figure 4).

To compare the mean data across different levels of each variable, we used multivariate analysis of variance MANOVA. This statistical test was applied to each independent variable to determine how the levels of the independent variable affect the dependent variables.

On day 4, Herculite Ultra XRV (Kerr, Italy) exhibited the largest difference among all three substances when immersed in Coca-Cola, with a mean of M = −9.02, *p* < 0.00. According to MANOVA, this indicates that the mean Δ value decreased by 9.02 for the composite kept in Coca-Cola for 4 days compared to the control group. By day 10, red wine caused the largest mean difference in ΔE, with M = 5.52, *p* < 0.00, signifying a decrease of 5.52 in the mean ΔE value for the composite kept in red wine for 10 days compared to the control group.

G-ænial A’CHORD (GC Corp, Japan), showed the most significant mean difference in ΔE when immersed in red wine on day 4, with M = 7.56, *p* < 0.00. According to MANOVA, this means the mean ΔE value decreased by 7.56 for the composite kept in red wine for 4 days compared to the control group, representing the largest difference among all three substances. On day 10, red wine continued to show the largest mean difference in ΔE, with M = 11.97, *p* < 0.00.

Omnichroma (Yamaguchi, Japan) experienced the largest difference among all three substances with coffee, which resulted in the most significant mean difference in ΔE on day 4, with M = 9.01, *p* < 0.00. According to MANOVA, this indicates a decrease of 9.01 in the mean ΔE value for the composite kept in coffee for 4 days compared to the control group. By day 10, coffee again showed the largest reported mean difference in ΔE, with M = 9.56, *p* < 0.00

In the comparison between the control group and the experimental groups exposed to different beverages for 10 days, significant positive differences were observed. For the Herculite Ultra XRV (Kerr, Italy) control and Herculite Ultra XRV (Kerr, Italy) immersed for 10 days in coffee pair, the control group’s mean increased from M = 0.63, SD ± 0.48 to M = 4.82, SD ± 1.30 in the experimental coffee group, with t = 14.37, *p* < 0.00 and a large effect size, Cohen’s d = 4.26. Similarly, for the Herculite Ultra XRV control and Herculite Ultra XRV (Kerr, Italy) immersed for 10 days in wine pair, the control group’s mean rose from M = 0.63, SD = ±0.48 to M = 5.95, SD = ±0.34 in the experimental wine group, with t = 66.97, *p* < 0.00 and an extremely strong effect size, Cohen’s d = 12.50. For the Herculite Ultra XRV (Kerr, Italy) control and Herculite Ultra XRV (Kerr, Italy) immersed for 10 days in cola pair, the control group’s mean increased from M = 0.638, SD = 0.48 to M = 3.99, SD = ±0.52 in the experimental cola group, with t = 29.75, *p* < 0.00 and a strong effect size, Cohen’s d = 6.64. These results indicate substantial increases in the delta value after continuous contact with coffee, wine, and cola, respectively (Table 4).

For the pair G-ænial A’CHORD (GC Corp, Japan), control and G-ænial A’CHORD (GC Corp, Japan), immersed for 10 days in coffee, there was a significant positive difference in mean ∆E, t = 79.81, *p* < 0.00, with the control group’s mean being M = 0.79, SD = ±0.53 and the experimental coffee group’s mean ∆E being M = 15.28, SD = ±0.74, resulting in a very strong effect size, Cohen’s d = 22.35. Similarly, for the G-ænial A’CHORD (GC Corp, Japan), control and G-ænial A’CHORD (GC Corp, Japan), immersed for 10 days in wine, a significant positive difference in mean ∆E is observed, t(14) = 125.91, *p* < 0.00, with the control group’s mean being M = 0.79, SD = 0.53 and the experimental wine group’s mean being M = 12.51, SD = ±0.38, indicating a very strong effect size, Cohen’s d = 25.02 (Table 5). For the pair G-ænial A’CHORD (GC Corp, Japan), control and G-ænial A’CHORD (GC Corp, Japan), immersed for 10 days in Coca-Cola, there is also a significant positive difference in mean ∆E, t = 66.81, *p* < 0.00, with the control group’s mean being M = 0.79, SD = ±0.53 and the experimental cola group‘s mean being M = 2.79, SD = ±0.16, showing a large effect size, Cohen’s d = 5.04.

For the pair Omnichroma (Yamaguchi, Japan) control and Omnichroma (Yamaguchi, Japan) immersed for 10 days in coffee, there was a significant positive difference in the mean delta, t = 40.61, *p* < 0.00, with the control group’s mean being M = 0.34, SD = ±0.31 and the experimental coffee group’s mean being M = 10.85, SD = ±1.03, resulting in a large effect size, Cohen’s d = 13.73. Similarly, for the pair Omnichroma (Yamaguchi, Japan) control and Omnichroma (Yamaguchi, Japan) immersed for 10 days in wine, a significant positive difference in the mean delta was observed, t = 75.01, *p* < 0.00, with the control group’s mean being M = 0.34, SD = 0.31 and the experimental wine group’s mean being M = 5.33, SD = 0.27, indicating a large effect size, Cohen’s d = 16.85 (Table 6). For the Omnichroma (Yamaguchi, Japan) control and Omnichroma (Yamaguchi, Japan) immersed for 10 days in Coca-Cola, there is also a significant positive difference in the mean delta, t = 11.16, *p* < 0.00, with the control group’s mean being M = 0.34, SD = ±0.31 and the experimental cola group’s mean being M = 1.28, SD = ±0.44, showing a large effect size, Cohen’s d = 2.43 (Table 6).

## 4. Discussion

The quality of dental composites is a critical factor that is intricately linked to clinical success. The adsorption of external colorants onto the surface and their absorption into the resin matrix can lead to color changes, compromising esthetic outcomes. Moreover, immersion in staining acidic solutions can significantly affect the hardness, roughness, flexural properties, and bond stability of restorative materials [20,21].

Numerous studies in the literature have examined the effects of resin materials on coloration after exposure to beverages like tea, coffee, and others. These studies have investigated the coloring impact of various substances, including tea, coffee, cola, wine, soy sauce, grape juice, chlorhexidine, vinegar, ayran, orange juice, and yogurt on composite resins. In our study, we used coffee, Coca-Cola, and red wine, aligning with these previous investigations. Our study’s findings are consistent with previous research highlighting the significant impact of beverages such as coffee, red wine, and Coca-Cola on color variations and surface integrity. These beverages cause substantial changes in both color and structure, emphasizing the importance of considering their effects [14].

This study examined the effects of immersing three different dental composites—Herculite Ultra XRV (Kerr, Italy), G-ænial A’CHORD (GC Corp, Japan), and Omnichroma (Yamaguchi, Japan)—in coffee, red wine, and Coca-Cola over a period of 10 days. The results of EDS analysis and SEM imaging revealed significant changes in both the surface morphology and elemental composition of the composites after immersion, providing insights into their degradation and discoloration behaviors. Immersion in different beverages resulted in notable changes in the elemental composition of Herculite Ultra XRV (Kerr, Italy). Carbon content decreased significantly in all three solutions, with red wine causing the most substantial reduction. Oxygen content also showed a marked decrease, particularly in composites immersed in red wine. Additionally, there was an increase in sulfur content and a significant increase in barium in Coca-Cola-immersed samples. SEM images supported these findings, showing extensive surface roughness and degradation, especially in samples immersed in Coca-Cola. The highest color change was observed with Coca-Cola after 4 days and with red wine after 10 days, indicating severe discoloration.

G-ænial A’CHORD (GC Corp, Japan), exhibited the highest susceptibility to coffee-induced degradation, with significant reductions in oxygen and aluminum content. Red wine also caused notable elemental changes, with significant decreases in carbon and silicon. SEM analysis showed increased surface roughness and porosity, particularly in the wine-immersed samples. The most significant color change was observed in coffee-immersed samples after 10 days, indicating substantial discoloration.

Omnichroma (Yamaguchi, Japan) demonstrated substantial surface and compositional changes after immersion in the beverages. The carbon content increased notably in Coca-Cola-immersed samples, reflecting significant organic residue deposition or chemical changes. Silicon content decreased sharply in samples immersed in Coca-Cola, highlighting severe degradation. SEM images showed pronounced surface degradation and erosion, especially in Coca-Colaimmersed samples. The most significant color change was observed in coffee-immersed samples after 10 days, indicating major discoloration.

Among the three composites, G-ænial A’CHORD (GC Corp, Japan), was the most affected by coffee immersion, showing the most significant reductions in oxygen and aluminum content. Herculite Ultra XRV (Kerr, Italy) was highly susceptible to red wine, with the largest reductions in carbon and oxygen. Omnichroma (Yamaguchi, Japan) experienced the most substantial changes in both carbon increase and silicon decrease when immersed in Coca-Cola, indicating severe compositional alterations and surface degradation.

The SEM images of the Herculite Ultra XRV (Kerr, Italy), G-ænial A’CHORD (GC Corp, Japan), and Omnichroma (Yamaguchi, Japan) composites reveal significant damage when exposed to different immersion solutions such as coffee, wine, and Coca-Cola, compared to control samples. Specifically, Herculite Ultra XRV (Kerr, Italy) showed the most damage when immersed in Coca-Cola, followed by wine and coffee, with the control sample exhibiting minimal damage. G-ænial A’CHORD (GC Corp, Japan) exhibited a similar pattern, with Coca-Cola causing the most damage, followed by substantial damage in wine and notable damage in coffee. Omnichroma (Yamaguchi, Japan) also displayed the greatest degradation in Coca-Cola, followed by wine and then coffee, with the control showing the least damage. In all cases, Coca-Cola caused the most severe damage, followed by wine and coffee, while the control samples had the fewest lesions.

The SEM analysis of these composites, compared with Filtek Z250 (3M ESPE), Filtek P90 (3M ESPE), Tetric Bulk Fill (Ivoclar Vivadent), SonicFill (Kerr), and Sinfony (3M ESPE) analyzed by Gezawi et al., highlights the significant impact of environmental conditions on material degradation [22]. Both sets of composites experienced notable surface deterioration under acidic conditions, with Coca-Cola causing the most pronounced damage.

In today’s age of highly esthetic dentistry, patients rightfully demand restorations that not only satisfy esthetic standards but also maintain their initial color and appearance over extended periods. Achieving proper color matching with the surrounding tooth tissue is paramount, not only initially but also for the restoration’s long-term durability.

In vitro comparisons of color stability are utilized as a method for predicting their degradability, particularly when supplemented by analysis at various levels of evaluation [22]. Although restorations in the oral cavity are subjected to a variety of dietary colorants and dynamic exposure to beverages, most studies involve exposing disks of the restorative material to a single coloring medium. Our findings revealed significant variations in color stability among the tested composites [22].

However, all color change values must be objectively assessed, as values of ΔE ≥ 3.7 are deemed clinically unacceptable. Surface changes in composite fillings due to common beverages add complexity to maintaining these esthetic standards. Variations in color parameters following immersion in red wine, black coffee, and Coca-Cola suggest that these substances adhere to the composite surfaces to varying degrees, leading to significant color alterations. The pronounced color changes observed with black coffee and red wine highlight the impact of these beverages on the composite materials. The higher ΔE value for black coffee compared to red wine underscores the more pronounced color alteration. Beltrami et al. found that when esthetic restorative materials with different finishing and polishing systems were immersed in a coffee solution for 28 days, nanofill composites exhibited the least color change, followed by nanohybrid and microhybrid composite resins. In a similar study on the color stability of composite resins, Topcu et al. reported that the microhybrid composite showed a higher level of color change compared to the nanofill composite [23].

Regarding the time of immersion of the resin composites specimenes, in a study by Çelik et al. (2016), samples were immersed in coffee, red wine, cola, and distilled water for 3 h daily, and their color was measured using spectrophotometers on the 1st, 7th, 15th, and 30th day [24].

In our study, samples made from a single composite resin were immersed in three different solutions coffee, Coca-cola and red wine. Initial color measurements were taken from the samples, which were then kept in these solutions for 10 days. Color measurements were subsequently recorded on the 4th and 10th day.

The composition of the resin matrix, as well as the type and size of the filler particles, significantly influence the color stability of composite resins. The resin matrix itself can be a key factor in the discoloration of composite resins. Various properties of the resin composition, including the chemical differences in resin monomers, the concentration and type of activators, initiators, and inhibitors, as well as the oxidation of unreacted monomers, have all been reported to affect the discoloration potential of composite resins [25].

The present study on dental composite addresses critical aspects of material degradation and color stability in the context of oral health, which is essential for therapeutic strategies and the long-term success of restorative dental treatments. This study evaluates the impact of commonly consumed beverages on the surface integrity and esthetic properties of modern dental composites, using advanced analytical techniques such as Scanning Electron Microscopy (SEM) and Energy-Dispersive X-ray Spectroscopy (EDS). These methods provide detailed insights into the mechanisms of material degradation and discoloration, contributing valuable knowledge to the development and optimization of biomaterials in dental applications. Furthermore, this study’s findings are pertinent to understanding how external factors influence the performance of biocompatible materials, aligning with the translational medical research, biomaterials, and therapeutic strategies. This research study not only advances the field of dental materials but also has broader implications for the design and application of composite resins in various biomedical contexts.

While this study provides valuable insights into the impact of common beverages on dental composites, it has several limitations. Firstly, in vitro conditions may not fully replicate the complex oral environment, where factors such as saliva flow, temperature variations, pH changes, and mechanical forces from chewing could further influence the degradation and discoloration of composites. Additionally, this study focused on only three types of composite resins and three beverages, which may not encompass the full range of materials and dietary habits encountered in clinical practice. The relatively short immersion period of 10 days, while useful for observing immediate effects, does not capture long-term changes that could occur over months or years. Additionally, the geometric shape of the fabricated specimens does not accurately reflect typical dental restorations Finally, this study did not account for the potential effects of brushing or other oral hygiene practices, which could mitigate the staining and degradation observed. Future research should consider these aspects to provide a more comprehensive understanding of how dietary and oral hygiene factors, including pH variations, influence the longevity and esthetics of dental composite restorations.

## 5. Conclusions

This study demonstrated that the tested beverages—coffee, red wine, and Coca-Cola—had a significant impact on the surface condition and color of the dental composite materials. SEM and EDS analyses revealed notable changes in surface morphology and elemental composition, highlighting the susceptibility of these composites to the acidic and staining properties of the beverages. Additionally, significant color changes were observed, indicating the extent to which these beverages can alter the esthetic appearance of the composites.

These findings underscore the importance of understanding the effects of common dietary beverages on dental composites. The varying degrees of degradation and discoloration highlight the need for careful material selection based on patient lifestyle and consumption habits.

## Figures and Tables

**Table 1:** Specifications of the materials used in this study.

Materials	Manufacturer	Classification	Resin Matrix	LOT
Herculite Ultra XRV	Kerr, Italy	Nanohybrid resin composite	Bis-GMA, TEGDMA	10198512
G-ænial A’CHORD	GC Corp, Tokyo, Japan	Hybrid resin composite	Bis-MEPP-based resin	230328C
Omnichroma	Tokuyama, omnich	Supra-nanohybrid resin composite	UDMA, TEGDMA	123E83

**Table 2:** Chemical changes in dental resin composites.

	Mass%	Initial SR (SD)	10 Days’ Immersion in Coffee (SD)	10 Days’ Immersion in Red Wine (SD)	10 Days’ Immersion in Coca-Cola (SD)
Herculite Ultra XRV (Kerr, Italy)	C	33.69 ± 2.32	21.68 ± 0.36	8.77 ± 0.23	10.68 ± 0.20
	O	40.16 ± 3.09	33.66 ± 0.50	8.15 ± 0.51	32.55 ± 0.37
	Al	10.71 ± 1.04	2.74 ± 0.11	1.60 ± 0.11	2.13 ± 0.09
	Si	15.43 ± 1.31	21.77 ± 0.31	9.27 ± 0.23	12.37 ± 0.19
	Ba		9.72 ± 0.39	8.34 ± 0.40	34.12 ± 0.58
	Ca		0.37 ± 0.06		0.53 ± 0.06
	Yb		10.05 ± 0.47		
	Cr			7.89 ± 0.34	
	Mn			0.46 ± 0.14	
	Fe			54.26 ± 1.07	
	S			1.28 ± 0.09	7.20 ± 0.14
	K				0.43 ± 0.05
G-ænial A’CHORD (GC Corp, Japan)	C	39.49 ± 2.20	34.82 ± 0.85	28.52 ± 0.54	53.09 ± 1.84
	O	31.89 ± 2.45	11.37 ± 0.70	44.98 ± 1.08	24.08 ± 1.99
	Al	6.08 ± 0.65	3.20 ± 0.26	1.35 ± 0.12	4.10 ± 0.45
	Si	22.53 ± 1.28	7.32 ± 0.38	6.34 ± 0.26	16.06 ± 0.89
	K		1.59 ± 0.22	0.81 ± 0.12	2.67 ± 0.47
	Ca		2.35 ± 0.28	11.27 ± 0.42	
	Fe		39.35 ± 1.81		
	SR			6.73 ± 0.53	
Omnichroma (Yamaguchi, Japan)	C	6.90 ± 0.22	27.14 ± 049	25.13 ± 0.55	65.44 ± 0.37
	O	43.16 ± 0.47	33.06 ± 0.58	25.55 ± 0.55	24.70 ± 0.48
	Na	0.70 ± 0.05	0.60 ± 0.06	0.36 ± 0.05	0.43 ± 0.48
	Si	36.36 ± 0.30	29.16 ± 0.33	37.55 ± 0.39	6.33 ± 0.13
	Zr	12.36 ± 0.27	9.16 ± 0.29	11.70 ± 0.35	1.55 ± 0.10
	Al	0.52 ± 0.04	0.36 ± 0.05		0.17 ± 0.03
	S				0.48 ± 0.04
	Ca				0.90 ± 0.07

**Table 3:** Comparison of the ΔE among the beverages and dental resins.

	Control Group Initial Color Mean (SD)	∆E 4 Days’ Immersion in Coffee (SD)	∆E 4 Days’ Immersion in Red Wine (SD)	∆ 4 Days’ Immersion in Coca-Cola (SD)	Control Group Initial Color Mean (SD)	∆E 10 Days’ Immersion in Coffee (SD)	∆E 10 Days’ Immersion in Red Wine (SD)	∆E 10 Days’ Immersion in Coca-Cola (SD)
Herculite Ultra XRV (Kerr, Italy)	0.64	8.31	5.40	9.66	0.43	4.83	5.96	4.00
	(±0.49)	(±2.53)	(±0.29)	(±0.28)	(±0.25)	(±1.30)	(±0.35)	(±0.52)
G-ænial A’CHORD (GC Corp, Japan)	0.79	5.83	8.35	3.06	0.54	15.28	12.51	2.80
	(±0.54)	(±0.28)	(±0.30)	(±0.31)	(±0.54)	(±0.74)	(±0.38)	(±0.16)
Omnichroma (Yamaguchi, Japan)	0.35	9.36	4.46	4.28	0.56	10.85	5.34	1.28
	(±0.32)	(±0.87)	(±0.30)	(±0.74)	(±0.33)	(±1.03)	(±0.28)	(±0.45)

**Table 4:** One-sample test for Herculite Ultra XRV (Kerr, Italy).

	Herculite Ultra XRV 4 Days	Herculite Ultra XRV 10 Days’ Immersion in Coffee	Herculite Ultra XRV 10 Days’ Immersion in Coca-Cola	Herculite Ultra XRV 10 Days’ Immersion in Red Wine
t	5.04	14.37	29.75	66.19
Mean Difference	0.63	4.82	3.99	5.95
*p*	0.00	0.00	0.00	0.00

**Table 5:** One-sample test for G-ænial A’CHORD (GC Corp, Japan).

	G-ænial A’CHORD 4 Days	G-ænial A’CHORD 10 Days’ Immersion in Coffee	G-ænial A’CHORD 10 Days’ Immersion in Coca-Cola	G-ænial A’CHORD 10 Days’ Immersion in Red Wine
t	5.67	79.81	66.81	125.91
Mean Difference	0.79	15.28	32.79	12.51
*p*	0.00	0.00	0.00	0.00

**Table 6:** One-sample test for Omnichroma (Yamaguchi, Japan).

	Omnichroma 4 Days	Omnichroma 10 Days’ Immersion in Coffee	Omnichroma 10 Days’ Immersion in Coca-Cola	Omnichroma 10 Days’ Immersion in Red Wine
t	4.25	40.61	11.16	75.01
Mean Difference	0.34	10.85	1.28	5.33
*p*	0.001	0.00	0.00	0.00

## Data Availability

The primary/raw data, from which the figures, graphs, and tables were generated, are available upon request from the corresponding author. These are the data we referred to for supplementary data, and they can be provided if requested. There are no ethical reasons restricting the data.
